# Clinical effectiveness of second-line antihyperglycemic drugs on major adverse cardiovascular events: An emulation of a target trial

**DOI:** 10.3389/fendo.2023.1094221

**Published:** 2023-01-30

**Authors:** Sukanya Siriyotha, Thitiya Lukkunaprasit, Teeranan Angkananard, Panu Looareesuwan, Gareth J. McKay, John Attia, Ammarin Thakkinstian

**Affiliations:** ^1^ Department of Clinical Epidemiology and Biostatistics, Faculty of Medicine Ramathibodi Hospital, Mahidol University, Bangkok, Thailand; ^2^ Department of Pharmacy Administration, College of Pharmacy, Rangsit University, Pathum Thani, Thailand; ^3^ Division of Cardiovascular Medicine, Department of Medicine, Faculty of Medicine, HRH Princess Maha Chakri Sirindhorn Medical Center, Srinakharinwirot University, Nakhon Nayok, Thailand; ^4^ Centre for Public Health, School of Medicine, Dentistry and Biomedical Sciences, Queen’s University Belfast, Belfast, United Kingdom; ^5^ Centre for Clinical Epidemiology and Biostatistics, School of Medicine and Public Health, Faculty of Health and Medicine, University of Newcastle, and Hunter Medical Research Institute, New Lambton, NSW, Australia

**Keywords:** diabetes, cardiovascular events, antihyperglycemic drugs, second-line, metformin

## Abstract

**Introduction:**

The cardiovascular benefits of multiple antihyperglycemic drugs as add-on therapies to metformin in the real-practice are unclear. This study aimed to directly compare major adverse cardiovascular events (CVE) associated with these multiple drugs.

**Methods:**

An emulation of a target trial was conducted using a retrospective-cohort data of type 2 diabetes mellitus (T2DM) prescribed with second-line drugs on top of metformin, including sodium-glucose cotransporter 2 inhibitors (SGLT2i), dipeptidyl peptidase-4 inhibitors (DPP4i), thiazolidinediones (TZD) and sulfonylureas (SUs). We applied inverse probability weighting and regression adjustment using intention-to-treat (ITT), per-protocol analysis (PPA) and modified ITT. Average treatment effects (ATE) were estimated using SUs as the reference.

**Results and Discussion:**

Among 25,498 patients with T2DM, 17,586 (69.0%), 3,261 (12.8%), 4,399 (17.3%), and 252 (1.0%) received SUs, TZD, DPP4i, and SGLT2i. Median follow-up time was 3.56 (1.36-7.00) years. CVE was identified in 963 patients. The ITT and modified ITT approaches showed similar results; the ATE (i.e., the difference of CVE risks) for SGLT2i, TZD, and DPP4i compared to SUs were -0.020(-0.040, -0.0002), -0.010(-0.017, -0.003), and -0.004(-0.010, 0.002), respectively, indicating 2% and 1% significant absolute risk reduction in CVE in SGLT2i and TZD compared to SUs. These corresponding effects were also significant in the PPA with ATEs of -0.045(-0.060, -0.031), -0.015(-0.026, -0.004), and -0.012(-0.020, -0.004). In addition, SGLT2i had 3.3% significant absolute risk reduction in CVE relative to DPP4i. Our study demonstrated benefits of SGLT2i and TZD in reducing CVE in T2DM patients compared to SUs when added to metformin.

## Introduction

Cardiovascular disease (CVD) is a major cause of disability and mortality among people with diabetes (1), with a two to three-fold (2) higher risk in these patients. Studies in middle-aged individuals with type 2 diabetes mellitus (T2DM) living in high- and middle-income countries showed that 27 out of 1,000 individuals died prematurely from CVD each year (3) with coronary artery disease and stroke identified as major contributors (2).

Recent meta-analyses (4–6) of a newer antihyperglycemic drug class [i.e., sodium-glucose cotransporter 2 inhibitors (SGLT2i): empagliflozin (7), canagliflozin (8), and dapagliflozin (9)] have demonstrated benefits in lowering fatal and non-fatal CVD, all-cause mortality, hospitalization with heart failure (HF), and declining kidney function in patients with T2DM (4), irrespective of glycemic control and baseline metformin and/or statin use (10). However, the advantages of SGLT2i in lowering major adverse cardiac events (MACE) were apparent only in those with established atherosclerotic CVD (ASCVD), particularly for patients with HF with both reduced ejection fraction (HFrEF) (11) and preserved ejection fraction (HFpEF) (12, 13), but less evident in patients with T2DM at greater risk of ASCVD (4, 8, 9). The 2021 European Society of Cardiology (ESC) guidelines on CVD prevention (14) advise clinicians to consider the use of SGLT2i in patients with T2DM without ASCVD, HF/HFrEF, or chronic kidney disease (CKD), based on their estimated future CVD risk. Previous studies have confirmed the benefits of SGLT2i as second-line oral antihyperglycemic agents that significantly reduce MACE and hospitalization with HF/HFrEF when compared to the use of other second-line therapies, including dipeptidyl peptidase-4 inhibitors (DPP4i) (5, 6, 15, 16) and sulfonylureas (SUs) (17).

To our knowledge, no direct comparisons of cardiovascular (CV) outcomes have been undertaken for second-line antihyperglycemic drug classes, including SGLT2i, DPP4i, SUs and thiazolidinediones (TZD). Several cohorts (18, 19) based on real-world studies data have compared SGLT2i with DPP4i, but those were mainly undertaken in western countries. Therefore, we conducted a cohort study using real-world practice data from Thai patients with T2DM, to directly compare CV outcomes following second-line oral antihyperglycemic agents as add-on therapies to metformin.

## Material and methods

**Figure 1:**
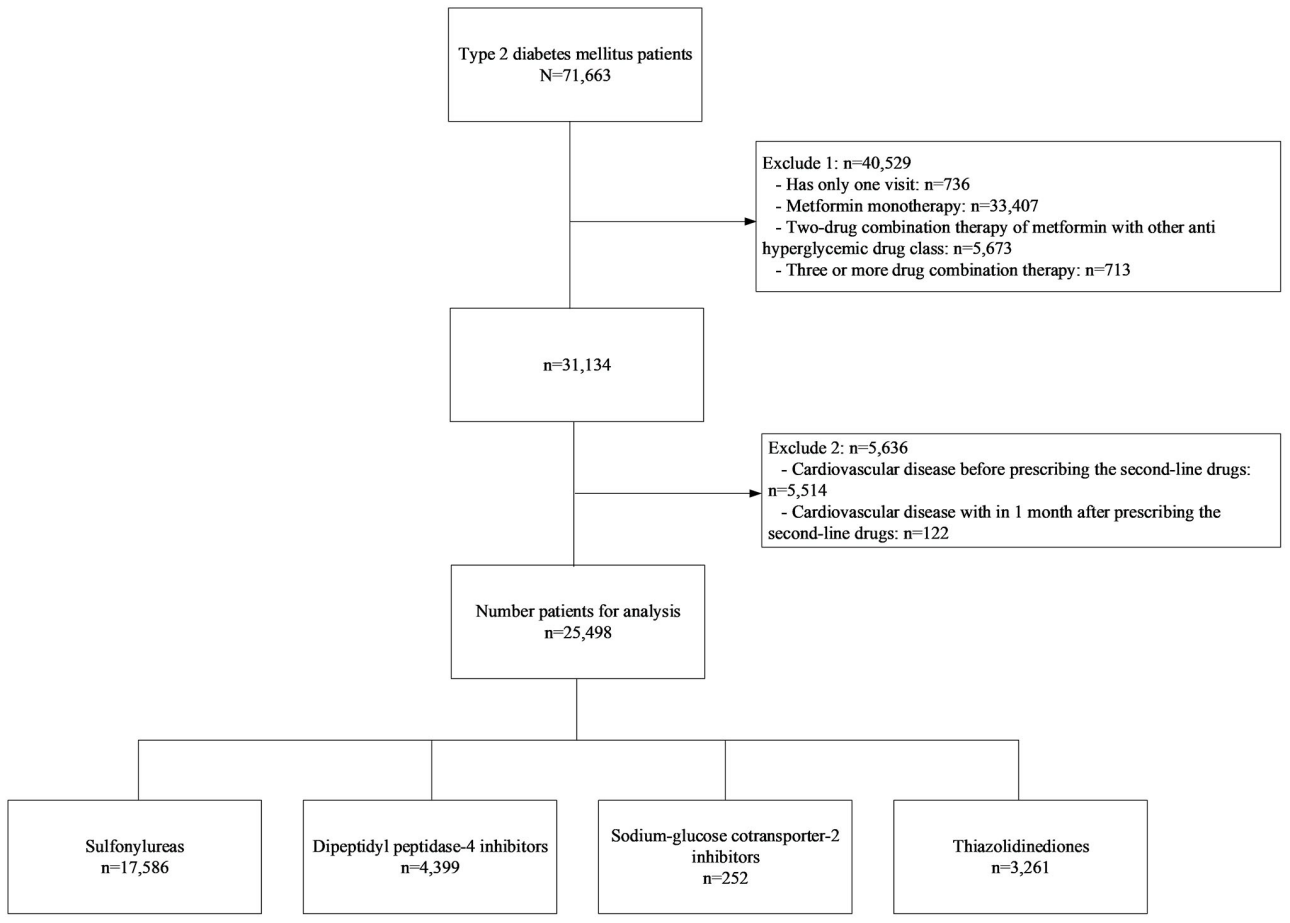
Flow of data for analysis.

A retrospective cohort study of T2DM was conducted at Ramathibodi Hospital, Bangkok, Thailand, from January 2010 to December 2019. Patients were included if they met the following criteria: adult patients aged 18 years or older diagnosed with T2DM from electronic databases by the International Statistical Classification of Diseases Ninth and Tenth Revisions (ICD-9 and ICD-10), and had prescription records of any of the following second-line antihyperglycemic drugs: SUs, TZD, DPP4i, or SGLT2i, added to metformin, see **Figure 1**. Participants with history of CVD events or reported CVD events within one month following the first prescription of a second-line treatment were excluded. The study was approved by the Institutional Review Board of the Faculty of Medicine, Ramathibodi Hospital, Mahidol University (COA. MURA2021/522).

Data were retrieved from electronic databases as follows: Baseline characteristics on receipt of second-line treatment included date, age, sex, body mass index, glycemic parameters (i.e., fasting blood glucose (FPG) and glycated hemoglobin (HbA1c)), renal function (i.e., estimated glomerular filtration rate (eGFR) based on the 2009 CKD-EPI creatinine equation), lipid profile (i.e., low-density lipoprotein cholesterol (LDL-C), high-density lipoprotein cholesterol (HDL-C), and triglycerides), underlying diseases (i.e., hypertension (HT), dyslipidemia (DLP), and CKD), and use of statin medication. Electronic medical records, laboratory data, diagnosis, and medication databases at baseline and follow up were retrieved. These data were then linked and merged to form a dynamic cohort dataset.

### Treatments and outcomes

Treatments of interest included SGLT2i, DPP4i, TZD, and SUs, which were identified from medication databases. All prescription data during follow up were retrieved including numbers of medication, daily dose/frequency, time to follow up, and time to ascertain medication use during the study period. The primary outcome of interest was time to CVD occurrence, which was identified by the ICD-10 (I20) codes including acute coronary syndrome, ischemic cardiomyopathy, chronic ischemic heart disease, HF/HFrEF, and cerebrovascular disease. The index date was the initial date for receipt of the second-line drug and the end-date was date at CVD occurrence or date of final visit if lost to follow up, or study end date if still CVD-free by December 31^st^ 2019. We emulated a target trial (20) using three analytical approaches: First, an intention-to-treat (ITT) approach considered the initial second-line drug regardless of whether treatment changed over time. Second, a per-protocol analysis (PPA) considered only patients who received a second-line treatment for the entire duration of the study period and did not switch treatments. Third, a modified ITT analysis censored patients at the time they switched treatments before they developed CVD.

### Statistical analysis

Frequency (percentage) and mean (SD) or median (range) were used to describe categorical and continuous variables, respectively. The data were compared between second-line drugs using Chi-squared or analysis of variance tests.

We applied treatment effect models with inverse probability weighting and regression adjustment (IPWRA) to estimate associations between each second-line treatment and CVD as follows:

First, the treatment model (TM) was constructed using a multinomial logit model by fitting treatment assignments to variables that were associated with treatment allocations (i.e., age, sex, BMI, FPG, HbA1c, HT, DLP, CKD, LDL-C, HDL-C, triglycerides, and statin use). Only significant variables were retained in the final TM, and a propensity score was estimated accordingly.

Second, the outcome model (OM) was constructed using a logit equation with an inverse probability weighting of treatment allocation (i.e., a propensity score) estimated from the TM. Significant variables associated with CVD were retained in the OM.

Third, the risk of CVD development in each second-line treatment group was estimated as a potential outcome mean (POM). An average treatment effect (ATE) along with 95% confidence intervals (CI) representing the difference between POMs, analogous to a risk difference, was estimated using SUs as the reference.

Finally, the TM model assumptions were assessed as follows: first, standardized mean differences [i.e., the difference of the mean covariates between two treatment groups divided by the standard deviation (21)] and the variance ratio [i.e., the ratio of two covariate variances (22)] were estimated. Then, these were weighted by inverse propensity score estimated from the TM. A TM was valid if the conditional independent assumption was achieved, i.e., weighted standardized mean differences for all covariates were less than 0.2 and the weighted variance ratios were close to 1, indicating that covariates were well balanced across treatment groups after weighting by inverse propensity score. Second, treatment overlap, or positive probability of receiving treatments, was assessed by plotting density of the probabilities for each second-line drug.

All analyses were performed using STATA version 17.0. (Stata Corp., College Station, TX, USA). A P-value < 0.05 was considered statistically significant.

## Results

A total of 71,663 patients with T2DM were identified, see **Figure 1**. Of these, 40,529 were excluded due to having only a single visit, receiving metformin monotherapy only or metformin with another treatment not considered of interest, or receiving triple combination therapy. In addition, a further 5,636 patients were excluded due to a recorded CVD event before receipt of a second-line treatment, or within one month of its prescription. Finally, 25,498 patients were included in the analysis which consisted of 17,586 (69.0%), 3,261 (12.8%), 4,399 (17.3%), and 252 (1.0%) who were in receipt of SUs, TZD, DPP4i, and SGLT2i, respectively.

### Baseline characteristics

Patient baseline characteristics are described in **Table 1**. The mean age and percentage female for each treatment group ranged from 57.1-64.9 years and 56.3%-59.6%, respectively. Before weighting by the inverse propensity score, many participant baseline characteristics included within the ITT approach were not balanced across treatment groups. For example, patients receiving SUs had poorer glycemic control and were more likely to be dyslipidemic. The majority of patients in receipt of DPP4i were older, hypertensive, with lower baseline eGFR, while those in receipt of SGLT2i were more likely to be obese.

**Table 1:** Baseline characteristics between second-line antihyperglycemic drugs.

Characteristics	SU	DPP4i	SGLT2i	TZD	P-value
	n=17,586	n=4,399	n=252	n=3,261	
Age, year, mean (SD)	61.2 (12.0)	64.9 (12.3)	57.1 (13.2)	60.1 (11.9)	<0.001
Sex, n (%)					
Female	10,277 (58.4)	2,623 (59.6)	150 (59.5)	1,835 (56.3)	0.030
Male	7,309 (41.6)	1,776 (40.4)	102 (40.5)	1,426 (43.7)	
BMI, kg/m^2^, mean (SD)	27.1 (4.5)	27.2 (4.8)	30.7 (5.8)	28.5 (5.2)	<0.001
FPG, mg/dL, mean (SD)	180.3 (79.4)	175.3 (87.4)	158.0 (50.3)	167.8 (75.4)	<0.001
HbA1c, %[mmol/mol], mean (SD)	8.2[66](1.8)	8.0[64](1.7)	7.8[62](1.5)	8.0[64] (1.7)	<0.001
eGFR, ml/min/1.73 m^2^, mean (SD)	73.6 (26.3)	69.3 (29.2)	88.6 (22.7)	75.0 (27.4)	<0.001
eGFR group, n (%)					
≥ 90 ml/min/1.73 m^2^	5,572 (31.7)	1,316 (29.9)	144 (57.1)	1,150 (35.3)	<0.001
60 – 89 ml/min/1.73 m^2^	6,473 (36.8)	1,491 (33.9)	73 (29.0)	1,165 (35.7)	
30 – 59 ml/min/1.73 m^2^	4,650 (26.5)	1,059 (24.1)	32 (12.7)	737 (22.6)	
15 – 29 ml/min/1.73 m^2^	602 (3.4)	336 (7.6)	3 (1.2)	141 (4.3)	
< 15 ml/min/1.73 m^2^	281 (1.6)	196 (4.5)	0 (0.0)	67 (2.1)	
HT, n (%)					
Yes	14,097 (80.2)	3,773 (85.8)	208 (82.5)	2,643 (81.0)	<0.001
No	3,489 (19.8)	626 (14.2)	44 (17.5)	618 (19.0)	
DLP, n (%)					
Yes	11,985 (68.2)	2,556 (58.1)	150 (59.5)	2,061 (63.2)	<0.001
No	5,601 (31.8)	1,843 (41.9)	102 (40.5)	1,200 (36.8)	
Statin, n (%)					
Yes	11,828 (67.3)	3,182 (72.3)	187 (74.2)	2,406 (73.8)	<0.001
No	5,758 (32.7)	1,217 (27.7)	65 (25.8)	855 (26.2)	
LDL-C, mg/dL, mean (SD)	118.6 (36.6)	112.1 (37.5)	113.1 (36.4)	115.7 (35.3)	<0.001
Triglycerides, mg/dL, median (range)	148.0 (118.0, 190.0)	143.0 (110.0, 190.0)	142.0 (114.9, 191.5)	140.0 (111.0, 178.9)	<0.001
HDL-C, mg/dL, mean (SD)	46.0 (10.9)	45.7 (11.8)	45.1 (10.6)	46.5 (11.0)	0.003

SU, sulfonylurea; DPP4i, dipeptidyl peptidase-4 inhibitors; SGLT2i, sodium-glucose cotransporter 2 inhibitors; TZD, thiazolidinedione; BMI, body mass index; CVD, cardiovascular disease; DLP, dyslipidemia; eGFR, estimated glomerular filtration rate; FPG, fasting plasma glucose; HDL-C, high density lipoprotein cholesterol; HT, hypertension; LDL-C, low density lipoprotein cholesterol.

The baseline characteristics of patients included in the PPA approach were similar to those included in the ITT analysis. However, there were significant differences between patients included and excluded in the PPA analysis, see **Supplementary Table 1**. Generally, those included tended to be older, had better FPG control, but poorer eGFR and were more likely to be hypertensive.

### Treatment effect model assumptions

Standardized mean differences and the variance ratios before and after weighting by the inverse propensity score are shown as raw and weighted values in **Table 2**. The absolute raw standardized mean differences ranged from 0.0020 to 0.3048, 0.0219 to 0.6727, and 0.0220 to 0.2742 for DPP4i vs. SUs, SGLT2i vs. SUs, and TZD vs. SUs, respectively. After weighting by inverse propensity score, the absolute weighted standardized mean differences of these corresponding comparisons were closer to zero, i.e., ranging from 0.0024 to 0.0275, 0.0172 to 0.1099, and 0.0036 to 0.0432, and the weighted variance ratios were close to one, indicating that covariates were well balanced. The balance plots and the density plots of the probabilities to receive each second-line drug also overlapped, see **Supplementary Figures 1, 2**. These suggested that the TM model assumptions hold, and the confounding variables across treatment groups were balanced successfully, providing a more correctly specified TM.

**Table 2:** Estimations of Standardized mean difference estimates for between treatment group factors before and after weighting by propensity score.

Estimation	Standardized differences		Variance ratio	
	Raw	Weighted	Raw	Weighted
DPP4i vs. SU				
Age, year	0.3048	-0.0275	1.0422	1.1447
Male vs. Female	-0.0233	0.0035	0.9917	1.0012
BMI, kg/m^2^	0.0020	0.0137	1.1004	1.0865
ln (FPG), mg/dL	-0.1058	-0.0218	1.0977	1.2008
HT	0.1489	-0.0059	0.7686	1.0095
eGFR < 60 ml/min/1.73 m^2^	0.0997	-0.0049	1.0707	0.9961
Statin medication	0.1108	-0.0024	0.9083	1.0020
HDL-C, mg/dL	-0.0285	0.0075	1.1814	1.2507
SGLT2i vs. SU				
Age, year	-0.3231	0.0570	1.2129	1.0493
Male vs. Female	-0.0219	0.0512	0.9959	1.0148
BMI, kg/m^2^	0.6727	0.0662	1.6404	1.0399
ln (FPG), mg/dL	-0.3412	0.0172	0.6618	0.9384
HT	0.0607	0.0485	0.9102	0.9214
eGFR < 60 ml/min/1.73 m^2^	-0.4297	-0.0325	0.5565	0.9738
Statin medication	0.1511	0.1099	0.8738	0.8998
HDL-C, mg/dL	-0.0788	0.0754	0.9530	1.3096
TZD vs. SU				
Age, year	-0.0975	-0.0080	0.9786	0.9882
Male vs. Female	0.0438	0.0080	1.0134	1.0027
BMI, kg/m^2^	0.2742	-0.0036	1.3312	0.9680
ln (FPG), mg/dL	-0.1991	0.0432	0.8994	1.2369
HT	0.0220	-0.0048	0.9667	1.0077
eGFR < 60 ml/min/1.73 m^2^	-0.0551	0.0115	0.9539	1.0089
Statin medication	0.1425	-0.0061	0.8790	1.0050
HDL-C, mg/dL	0.0523	-0.0053	1.0254	1.0259

BMI, body mass index; CVD, cardiovascular disease; DLP, dyslipidemia; eGFR, estimated glomerular filtration rate; FPG, fasting plasm glucose; HDL-C, high density lipoprotein cholesterol; HT, hypertension; LDL-C, low density lipoprotein cholesterol.

### CVD events, TM, and OM

The median follow-up time was 3.56 (IQR: 1.36-7.00) years. Of the 25,498 patients, 963 had a recorded CVD event representing an incidence (95% CI) of 3.8% (3.5%, 4.0%). Most of CVD events were ischemic heart disease (64.4%) followed by cerebrovascular disease (17.6%), see **Supplementary Table 2.** The CVD incidence (95% CI) for patients taking SGLT2i, TZD, SUs, and DPP4i was 2.4% (0.9%, 5.1%), 2.9% (2.3%, 3.5%), 3.9% (3.6%, 4.2%), and 4.0% (3.5%, 4.6%), respectively. Factors significantly associated with treatment assignment and retained in the final TM to estimate the propensity score included age, sex, BMI, FPG, HT, eGFR, HDL-C, and statin use, see **Supplementary Table 3**. Similarly, factors significantly associated with CVD development in the OM were age, sex, BMI, HT, eGFR, HDL-C, and statin use (data not shown). Both TM and OM were considered in the IPWRA doubly robust approach, and the results are shown in **Tables 3**, **4**. For the ITT approach, the POMs (95% CI) in the SGLT2i, TZD, DPP4i, and SUs groups were 0.020 (0.001, 0.040), 0.030 (0.024, 0.037), 0.036 (0.031, 0.041), and 0.040 (0.037, 0.043), respectively, indicating the risk of CVD development of 2%, 3%, 3.6%, and 4% in the corresponding treatment groups (**Table 3**). The ATEs representing the risk difference in the POMs between SGLT2i, TZD, DPP4i vs. SUs were -0.020 (-0.040, -0.0002), -0.010 (-0.017, -0.003), and -0.004 (-0.010, 0.002), respectively, see values above the diagonal line in **Table 4**, indicating 2% and 1% absolute CVD risk reductions associated with SGLT2i and TZD, when compared to the more commonly prescribed SU group. The comparison of the ATE between SGLT2i and TZD was not significantly different [ATE of -0.010 (-0.031, 0.010)]. In addition, relative risk (RR) along with 95% CIs were estimated by dividing POM of each treatment by POM of a reference treatment, see values underneath the diagonal line in **Table 4**. The RRs compared to SUs were 0.50 (0.01, 0.99), 0.76 (0.59, 0.93), and 0.89 (0.74, 1.04) for those receiving SGLT2i, TZD, and DPP4i, respectively, indicating that a relative risk reduction for CVD was 50% and 24% in patients who received SGLT2i and TZD, respectively, when compared to SUs. The RR of SGLT2i relative to DPP4i was 0.56 (0.01, 1.12), but this was not significant.

**Table 3:** Estimation of Potential outcome mean estimates between second-line drugs: Treatment effect model with inverse probability weighting and regression adjustment.

Treatment	POM	Lower limit	Upper limit
ITT			
DPP4i	0.036	0.031	0.041
SGLT2i	0.020	0.001	0.040
TZD	0.030	0.024	0.037
SU	0.040	0.037	0.043
PPA			
DPP4i	0.046	0.039	0.053
SGLT2i	0.013	0.0001	0.026
TZD	0.043	0.033	0.053
SU	0.058	0.054	0.063
Modify ITT			
DPP4i	0.036	0.031	0.041
SGLT2i	0.020	0.001	0.040
TZD	0.030	0.024	0.037
SU	0.040	0.037	0.043

POM, Potential outcome mean; ITT, intention-to-treat; PPA, per-protocol analysis; DPP4i, dipeptidyl peptidase-4 inhibitors; SGLT2i, sodium-glucose cotransporter 2 inhibitors; TZD, thiazolidinedione; SU, sulfonylurea.

**Table 4:** Relative treatment effect estimates between second-line drugs: Treatment effect model with inverse probability weighting and regression adjustment.

**RR** ↓	**ATE** →				
	Treatment	SU	DPP4i	SGLT2i	TZD
	**ITT**				
	SU	ref	-0.004 (-0.010, 0.002)	-0.020 (-0.040, -0.0002)	-0.010 (-0.017, -0.003)
	DPP4i	0.89 (0.74, 1.04)	ref	-0.016 (-0.036, 0.005)	-0.005 (-0.014, 0.003)
	SGLT2i	0.50 (0.01, 0.99)	0.56 (0.01, 1.12)	ref	0.010 (-0.010, 0.031)
	TZD	0.76 (0.59, 0.93)	0.85 (0.63, 1.07)	1.51 (0.01, 3.01)	ref
	**PPA**				
	SU	ref	-0.012 (-0.020, -0.004)	-0.045 (-0.060, -0.031)	-0.015 (-0.026, -0.004)
	DPP4i	0.78 (0.65, 0.93)	ref	-0.033 (-0.048, -0.018)	-0.003 (-0.015, 0.010)
	SGLT2i	0.22 (0.001, 0.45)	0.28 (0.01, 0.58)	ref	0.031 (0.014, 0.047)
	TZD	0.74 (0.56, 0.92)	0.94 (0.68, 1.21)	3.37 (0.001, 6.97)	ref
	**Modify-ITT**				
	SU	ref	-0.004 (-0.010, 0.002)	-0.020 (-0.040, -0.0002)	-0.010 (-0.017, -0.003)
	DPP4i	0.89 (0.74, 1.04)	ref	-0.016 (-0.036, 0.005)	-0.005 (-0.014, 0.003)
	SGLT2i	0.50 (0.01, 0.99)	0.56 (0.01, 1.12)	ref	0.010 (-0.010, 0.031)
	TZD	0.76 (0.59, 0.93)	0.85 (0.63, 1.07)	1.51 (0.01, 3.01)	ref

ATE, Average treatment effects or risk difference in cells above diagonal line, each comparison pair is read from right to left; RR, Relative risk ratio in cell under a diagonal line,each comparison pair is read from left to right; SU, sulfonylurea; DPP4i, dipeptidyl peptidase-4 inhibitors; SGLT2i, sodium-glucose cotransporter 2 inhibitors; TZD, thiazolidinedione.

For the PPA approach, the POMs in the SGLT2i, TZD, DPP4i, and SUs groups were 0.013 (0.0001, 0.026)), 0.043 (0.033, 0.053), 0.046 (0.039, 0.053), and 0.058 (0.054, 0.063), respectively. SGLT2i, TZD, and DPP4i were significantly associated with a 4.5%, 1.5%, and 1.2% reduced CVD risk (representing ATEs of -0.045 (-0.060, -0.031), -0.015 (-0.026, -0.004), and -0.012 (-0.020, -0.004), respectively) when compared to SUs. In addition, SGLT2i were significantly associated with a 3.3% lower CVD risk when compared to DPP4i (ATE: -0.033 (-0.048, -0.018)). The results from the modified ITT were similar to those from the ITT because none of patients switched their second-line treatments before CVD development.

Relative risks (RR) with 95% CI converted from the ATEs are also shown in **Table 4**. Those receiving SGLT2i, TZD, and DPP4i had 0.50 (0.01, 0.99), 0.76 (0.59, 0.93), and 0.89 (0.74, 1.04) times lower CVD risk than SUs but only SGLT2i and TZD were significant. In addition, the SGLT2i group had 0.56 (0.01, 1.12) times lower CVD risk than DPP4, but this was not significant.

## Discussion

To our knowledge, this is the first emulated target trial based on real-world practice data that directly compared CV outcomes associated with second-line antihyperglycemic medications to the low-cost SU drug class, when added to metformin therapy in a Southeast Asian cohort. During the 42-month follow-up period, CVD risk associated with both SGLT2i and TZD treatment options were significantly reduced relative to the SUs drug class across all three approaches. These added benefits were also significant with the DPP4i group when evaluated using the PPA approach.

The findings of this study support other real-world trials (15, 23) highlighting the potential benefit of lower CVD risk associated with SGLT2i when prescribed following metformin therapy. However, no studies have emulated a target trial by directly investigating the effects of second-line drugs and comparing the outcomes to the more commonly prescribed SUs using analytical approaches that imitate an RCT. Notably, TZDs (mainly pioglitazone) have shown desirable effects in reducing CVD risk, when added to metformin (24). Moreover, the lack of a significant reduction in CVD risk associated with DPP4i in the ITT approach may have resulted from changing medication in the follow-up period and similarly, for SGLT2i compared to DPP4i in the PPA approach (**Table 4**), a comparison which has not been previously described.

The effects of SGLT2i are less powerful on the prevention of ASCVD when compared with the prevention of HF and renal outcomes. The possible mechanisms of action are its ameliorating effects on atherogenesis and established CV risk factors, i.e., weight reduction associated with glucosuria (25), blood pressure lowering by natriuresis and a diuretic effect (26), reduction of albuminuria, inflammation and oxidative markers (27), improving vascular compliance, and endothelial function by attenuating endothelial cell activation, inducing direct vasorelaxation, and reducing endothelial cell dysfunction (28, 29). In addition, the CV benefits, particularly HF and CV-related mortality, may arise as a consequence of renal effects and reduced intraglomerular pressure that leads to renal protection, improved renal function and/or reduced renal stress; this may indirectly improve cardiac function through various pathways, including reduced reactive oxygen species generation, afferent sympathetic nervous system activation, and inflammation (30). Moreover, SGLT2i can improve mitochondrial respiratory function and cardiac energy metabolism in the failing heart (31) when mitochondrial glucose oxidation and energy production is diminished. Extrapolating to the population level, we previously found that SGLT2i could directly lower CKD risk about 14% (32), and in this study, they could lower CVD risk of 2% relative to SUs with a number needed to treat of 140 and 20 per 1000 treated patients, i.e., for each 100 patients treated, we would prevent 14 CKD events and 2 CVD events over ~3.5 years of follow. Our current data also suggested that CKD patients had about 1.71 higher odds of CVD occurrence compared to those free from CKD. Based on mediation analysis (33), an indirect effect of SGLT2i on CVD risk through lowering CKD can be estimated by the product method [i.e., exponential [(-0.14) x ln(1.71)], and indicates a 0.92 odds of reduced CVD risk from lowering CKD, see **Supplementary Figure 3**.

The beneficial effects of CVD risk reduction associated with pioglitazone was previously investigated in patients with insulin resistance, pre-diabetes and T2DM (34). The underlying mechanism may be due to delayed atheroma progression by reducing the ratio of triglyceride/high-density lipoprotein-cholesterol (35) and increasing cholesterol efflux capacity (36) which has been reported to be inversely associated with the incidence of CV events in a population-based cohort (37). Similarly, apart from the glucose-lowering effect, DPP4i may also exert a positive influence on CVD and CV risk factors given its reported modest beneficial effects on postprandial lipemia, body weight, blood pressure, inflammatory markers, oxidative stress, and endothelial function in patients with T2DM (38).

DPP4i influence enzyme expression through the cell surface receptors that primarily effect the heart, and are mainly mediated by the products of stromal cell-derived factor-1, a stem cell chemokine that promotes inflammation, regeneration and repair (39). It can cause significant deleterious effects to cardiomyocytes in states of diabetes and other cardiac stress (40). However, DPP4i offer positive inotropic effects by enhancing the actions of glucagon like peptide-1 to stimulate cyclic AMP in cardiomyocytes. Previous pooled analyses demonstrated that DPP4i do not increase the risk of HF in patients with T2DM and a previous history of HF, but should be used with caution especially in patients with established ASCVD and no history of HF (41).

In our setting, SUs were commonly used as second-line drugs, a treatment modality similar to other countries in resource limited settings where more recent second-line medications are more expensive, unavailable or not reimbursable in the Universal Health Coverage in Thailand. Considering the CV effects associated with SUs, recent evidence suggests no difference in MACE outcomes in T2DM patients at high CV risk, relative to modern agents such as gliclazide MR and glimepiride (42–44). International and national guidelines still recommend using SUs (with the exception of glibenclamide), an alpha-glucosidase inhibitor or a DPP4i for dual therapy after metformin failure (45). Recently, the GRADE study (46) was conducted to assess treatment efficacy of insulin glargine, glimepiride (SUs), liraglutide (glucagon-like peptide-1 receptor agonists [GLP-1 RA]), and sitagliptin (DPP4i) on microvascular and CVD outcomes when added to metformin. This study found that adding liraglutide could significantly lower the risk of any CVD about 29% relative to other three combined treatments [hazard ratio = 0.71 (95% CI: 0.56, 0.90)]. However, SGLT2i was not considered in this study, and our study did not assess GLP1-RA due to insufficient data. Both treatments may be beneficial for lowering CV events in T2DM patients, in which the choice of second-line oral antihyperglycemic agents should be individualized based on the patient’s clinical needs, preference, and economic constraints, particularly in countries with limited resources.

Our study had several strengths. This study was the first analysis of second-line antihyperglycemic medications for CVD risk reduction in a large-scale population with T2DM based on real-world practice. We also applied three analytical approaches (ITT, PPA and modified ITT) to emulate a target trial on our real-world data. We evaluated the role of newer second-line agents compared to the more commonly used approach of SUs which are still the most commonly prescribed medication in resource limited countries. Furthermore, pioglitazone, which was the only TZD still available and commonly prescribed, was found to provide similar protective benefits as SGLT2i, which would support their continued use in real-world practice.

This study had some limitations. Data used in this study was based on a single center which may limit its generalizability. Only a small number of patients received SGLT2i in Ramathibodi Hospital since its release in 2015. As such, the estimated relative treatment effect is imprecise. Likewise, similarly limited data for GLP-1 RA were available, and none of the patient developed CVD, therefore we were unable to estimate its relative treatment effect. Further multi-center studies are needed to explore the potential benefits of CVD risk reduction associated with both SGLT2i and GLP-1 RA prescribed as an add-on to metformin in those with T2DM. In addition, different drug dosages of individual drugs were not evaluated and should be further explored. Finally, neither CV nor all-cause mortality was considered because none of patients died in the SGLT2i group. These and other important outcomes including HFrEF and peripheral artery disease should also be further considered in longer follow up period.

## Conclusion

Our study demonstrated the potential benefits associated with SGLT2i and TZD (pioglitazone) in reducing the risk of CVD in T2DM patients compared to the more commonly prescribed SUs, in real-world practice data. Further multi-center studies should be performed to evaluate GLP-1 RA following metformin treatment to assess its effects compared to other second-line antihyperglycemic agents.

## Data availability statement

The raw data supporting the conclusions of this article will be made available by the authors, without undue reservation.

## Ethics statement

The studies involving human participants were reviewed and approved by Institutional Review Board of the Faculty of Medicine, Ramathibodi Hospital, Mahidol University (COA. MURA2021/522). The patients/participants provided their written informed consent to participate in this study.

## Author contributions

SS contributed substantially to conception and study design, acquisition, analysis, and interpretation of the data, and drafted the article. TL contributed substantially to analysis and interpretation of the data, and drafted the article. TA contributed substantially to data interpretation, drafted and revised the article. PL contributed substantially to data acquisition, analysis, and revised the article. GJM, and JA contributed substantially to data interpretation and revised the article. AT contributed substantially to conception, study design, analysis and interpretation of the data, and revised the article. All authors contributed to the article and approved the submitted version.
