# Supplementary material for: Clinical effectiveness of second-line antihyperglycemic drugs on major adverse cardiovascular events: An emulation of a target trial
Source: Front Endocrinol (Lausanne). 2023 Jan 30;14:1094221. doi: 10.3389/fendo.2023.1094221 (PMC9922758; doi:10.3389/fendo.2023.1094221)
Supplement: Supplementary file 1 [file DataSheet_1.docx]

**Supplementary Legend**

Supplementary Figure 1 Balance plots of variables associated with second-line drug

allocation

Supplementary Figure 2 Density plots for the probabilities to receive each second-line

drug

Supplementary Figure 3 Extrapolating the effect of SGLT2i on reduced CVD risk from lowering CKD based on the concept of medication analysis

Supplementary Table 1 Comparison of patient characteristics in receipt of second-line anti-

hyperglycemic drugs included and excluded in the PPA approach

Supplementary Table 2 Types of cardiovascular events among 963 patients

Supplementary Table 3 Factors associated with second-line drug allocation: A

multivariate multinomial logistic regression

Supplementary Figure 1. Balance plots of variables associated with second-line drug allocation

^BMI: body mass index; FPG: Fasting plasma glucose; eGFR: Estimated glomerular filtration rate;^ ^HDL-C: High density lipoprotein cholesterol; SU: sulfonylurea; DPP4i: dipeptidyl peptidase-4 inhibitors; SGLT2i: sodium-glucose cotransporter 2 inhibitors; TZD: thiazolidinediones^

Supplementary Figure 2. Density plots for the probabilities to receive each second-line drug

^SU: sulfonylurea; DPP4i: dipeptidyl peptidase-4 inhibitors; SGLT2i: sodium-glucose cotransporter 2 inhibitors; TZD: thiazolidinediones^

Supplementary Figure 3. Extrapolating the effect of SGLT2i on reduced CVD risk from lowering

CKD based on the concept of medication analysis


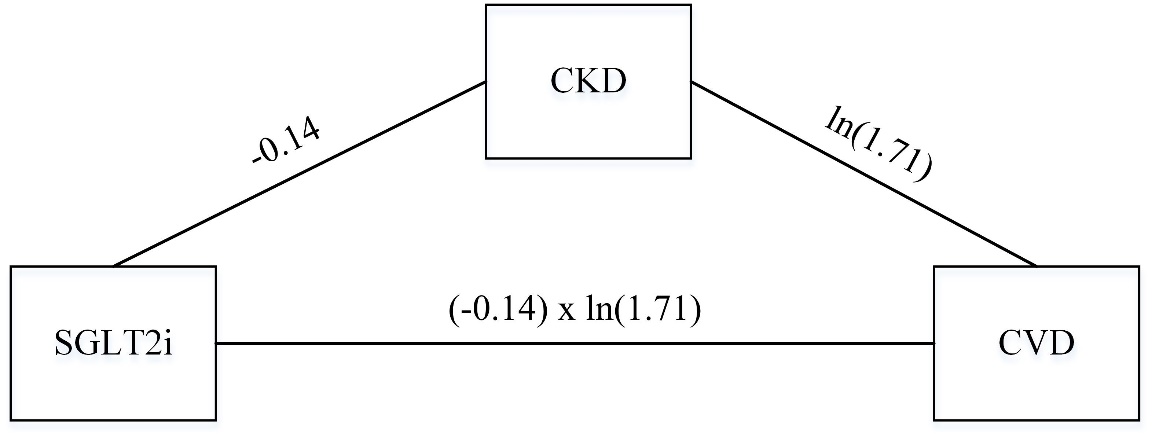


^CKD: chronic kidney disease; CVD: cardiovascular disease; SGLT2i: sodium-glucose cotransporter 2 inhibitors^

Supplementary Table 1. Comparison of patient characteristics in receipt of second-line anti-hyperglycemic drugs included and excluded in the PPA approach

| Characteristics | PPA | | | | Excluded from PPA | | | |
| --- | --- | --- | --- | --- | --- | --- | --- | --- |
|  | SU | DPP4i | SGLT2i | TZD | SU | DPP4i | SGLT2i | TZD |
|  | n=11,225 | n=2,999 | n=177 | n=1,955 | n=6,361 | n=1,400 | n=75 | n=1,306 |
| Age, year, mean (SD) | 62.4 (12.0) | 66.5 (12.0) | 57.8 (12.8) | 60.8 (12.2) | 59.0 (11.7) | 61.4 (12.1) | 55.5 (14.1) | 58.9 (11.3) |
| Gender, n (%) |  |  |  |  |  |  |  |  |
| Female | 6,411 (57.1) | 1,771 (59.1) | 104 (58.8) | 1,080 (55.2) | 3,866 (60.8) | 852 (60.9) | 46 (61.3) | 755 (57.8) |
| Male | 4,814 (42.9) | 1,228 (40.9) | 73 (41.2) | 875 (44.8) | 2,495 (39.2) | 548 (39.1) | 29 (38.7) | 551 (42.2) |
| BMI, kg/m^2^, mean (SD) | 26.7 (4.3) | 26.9 (4.7) | 30.6 (5.7) | 28.2 (5.2) | 27.9 (4.8) | 27.8 (4.8) | 30.9 (6.2) | 28.9 (5.2) |
| FPG, mg/dL, mean (SD) | 177.9 (82.1) | 173.3 (92.1) | 155.1 (50.8) | 164.5 (77.9) | 184.3 (74.2) | 179.5 (76.1) | 164.9 (48.7) | 172.7 (71.4) |
| HbA1c, %, mean (SD) | 8.0 (1.8) | 7.8 (1.7) | 7.7 (1.6) | 7.9 (1.7) | 8.4 (1.8) | 8.3 (1.7) | 7.9 (1.4) | 8.3 (1.7) |
| eGFR,ml/min/1.73m^2^,mean(SD) | 71.6 (26.6) | 66.8 (29.4) | 88.3 (22.6) | 74.1 (28.2) | 77.2 (25.3) | 74.7 (28.1) | 89.2 (22.8) | 76.3 (26.0) |
| eGFR group, n (%) |  |  |  |  |  |  |  |  |
| ≥ 90 ml/min/1.73 m^2^ | 3,272 (29.2) | 814 (27.2) | 101 (57.1) | 700 (35.8) | 2,300 (36.2) | 502 (35.9) | 43 (57.3) | 450 (34.5) |
| 60 – 89 ml/min/1.73 m^2^ | 4,056 (36.2) | 1,015 (33.9) | 52 (29.4) | 643 (32.9) | 2,417 (38.0) | 476 (34.0) | 21 (28.0) | 522 (40.0) |
| 30 – 59 ml/min/1.73 m^2^ | 3,239 (28.9) | 749 (25.0) | 22 (12.4) | 474 (24.2) | 1,411 (22.2) | 310 (22.1) | 10 (13.3) | 263 (20.2) |
| 15 – 29 ml/min/1.73 m^2^ | 434 (3.9) | 258 (8.6) | 2 (1.1) | 88 (4.5) | 168 (2.6) | 78 (5.6) | 1 (1.3) | 53 (4.1) |
| < 15 ml/min/1.73 m^2^ | 217 (1.9) | 162 (5.4) | 0 (0.0) | 50 (2.6) | 64 (1.0) | 34 (2.4) | 0 (0.0) | 17 (1.3) |
| HT, n (%) |  |  |  |  |  |  |  |  |
| Yes | 9,139 (81.4) | 2,611 (87.1) | 148 (83.6) | 1,616 (82.7) | 4,958 (77.9) | 1,162 (83.0) | 60 (80.0) | 1,027 (78.6) |
| No | 2,086 (18.6) | 388 (12.9) | 29 (16.4) | 339 (17.3) | 1,403 (22.1) | 238 (17.0) | 15 (20.0) | 279 (21.4) |
| DLP, n (%) |  |  |  |  |  |  |  |  |
| Yes | 7,707 (68.7) | 1,719 (57.3) | 100 (56.5) | 1,243 (63.6) | 4,278 (67.3) | 837 (59.8) | 50 (66.7) | 818 (62.6) |
| No | 3,518 (31.3) | 1,280 (42.7) | 77 (43.5) | 712 (36.4) | 2,083 (32.7) | 563 (40.2) | 25 (33.3) | 488 (37.4) |
| Statin, n (%) |  |  |  |  |  |  |  |  |
| Yes | 7,157 (63.8) | 2,156 (71.9) | 132 (74.6) | 1,393 (71.3) | 4,671 (73.4) | 1,026 (73.3) | 55 (73.3) | 1,013 (77.6) |
| No | 4,068 (36.2) | 843 (28.1) | 45 (25.4) | 562 (28.7) | 1,690 (26.6) | 374 (26.7) | 20 (26.7) | 293 (22.4) |
| LDL-C, mg/dL, mean (SD) | 118.8 (36.9) | 111.2 (37.6) | 110.7 (34.2) | 116.4 (37.0) | 118.3 (36.0) | 114.0 (37.1) | 118.7 (40.8) | 114.7 (32.6) |
| Triglycerides, mg/dL, median (range) | 148.7  (119.0, 187.0) | 141.3  (108.7, 186.0) | 141.0  (111.0, 192.0) | 141.0  (111.2, 179.1) | 146.6  (117.3, 197.0) | 148.0  (112.0, 200.0) | 146.0  (123.0, 191.0) | 139.0  (110.8, 177.4) |
| HDL-C, mg/dL, mean (SD) | 46.0 (10.7) | 46.1 (12.0) | 45.7 (10.9) | 46.5 (10.9) | 45.9 (11.1) | 44.8 (11.2) | 43.8 (9.7) | 46.5 (11.1) |
| ^BMI: body mass index; HbA1C: Hemoglobin A1C; DLP: Dyslipidemia; eGFR: Estimated glomerular filtration rate;^ ^FPG: Fasting plasma glucose; HDL-C: High density lipoprotein cholesterol; HT: Hypertension; LDL-C: Low density lipoprotein cholesterol^ | | | | | | | | |

Supplementary Table 2. Types of cardiovascular events among 963 patients

| Types of cardiovascular events | n (%) |
| --- | --- |
| Ischemic heart disease | 617 (64.07) |
| Cerebrovascular disease | 169 (17.55) |
| Acute coronary syndrome | 89 (9.24) |
| Heart failure | 72 (7.48) |
| Unspecified | 16 (1.66) |

Supplementary Table 3. Factors associated with second-line drug allocation: A multivariate

multinomial logistic regression

| Factors | OR (95% CI) | P-value |
| --- | --- | --- |
| DPP4i vs. SU |  |  |
| Age, year | 1.028 (1.024, 1.031) | <0.001 |
| Male vs. Female | 1.010 (0.941, 1.084) | 0.783 |
| BMI, kg/m^2^ | 1.019 (1.011, 1.027) | <0.001 |
| ln (FPG), mg/dL | 0.854 (0.771, 0.945) | 0.002 |
| HT | 1.146 (1.039, 1.263) | 0.007 |
| eGFR < 60 ml/min/1.73 m^2^ | 0.931 (0.862, 1.005) | 0.065 |
| Statin | 1.178 (1.093, 1.270) | <0.001 |
| HDL-C, mg/dL | 0.996 (0.992, 0.999) | 0.008 |
| SGLT2i vs. SU |  |  |
| Age, year | 0.991 (0.980, 1.004) | 0.166 |
| Male vs. Female | 1.008 (0.771, 1.316) | 0.956 |
| BMI, kg/m^2^ | 1.109 (1.086, 1.132) | <0.001 |
| ln (FPG), mg/dL | 0.279 (0.179, 0.433) | <0.001 |
| HT | 1.096 (0.776, 1.547) | 0.604 |
| eGFR < 60 ml/min/1.73 m^2^ | 0.378 (0.259, 0.551) | <0.001 |
| Statin | 1.330 (0.995, 1.778) | 0.054 |
| HDL-C, mg/dL | 0.994 (0.982, 1.007) | 0.368 |
| TZD vs. SU |  |  |
| Age, year | 0.996 (0.993, 1.000) | 0.050 |
| Male vs. Female | 1.191 (1.100, 1.290) | <0.001 |
| BMI, kg/m^2^ | 1.057 (1.049, 1.066) | <0.001 |
| ln (FPG), mg/dL | 0.526 (0.465, 0.595) | <0.001 |
| HT | 0.955 (0.863, 1.058) | 0.377 |
| eGFR < 60 ml/min/1.73 m^2^ | 0.936 (0.856, 1.024) | 0.150 |
| Statin | 1.318 (1.209, 1.436) | <0.001 |
| HDL-C, mg/dL | 1.008 (1.004, 1.011) | <0.001 |

^BMI: body mass index; eGFR: Estimated glomerular filtration rate; FPG: Fasting plasma glucose; HDL-C: High density lipoprotein cholesterol; HT: Hypertension; LDL-C: Low density lipoprotein cholesterol^
